# Mannose-Binding Lectin: A Potential Therapeutic Candidate against* Candida* Infection

**DOI:** 10.1155/2018/2813737

**Published:** 2018-05-02

**Authors:** Noha M. Hammad, Nissreen E. El Badawy, Hamed A. Ghramh, Laila M. Al Kady

**Affiliations:** ^1^Department of Medical Microbiology and Immunology, Faculty of Medicine, Zagazig University, Zagazig, Egypt; ^2^Research Center for Advanced Materials Science (RCAMS), King Khalid University, P.O. Box 9004, Abha 61413, Saudi Arabia; ^3^Department of Biology, Faculty of Science, King Khalid University, P.O. Box 9004, Abha 61413, Saudi Arabia

## Abstract

Mannose-binding lectin (MBL) is one of the key players in the innate immune system. It has the ability to identify a broad range of pathogens based on recognition of carbohydrate repeats displayed on microbial surfaces. Since mannans make about 40% of the total polysaccharide content of cell wall of* Candida *species (spp.) and MBL is capable of high-affinity binding to the mannan fraction of their cell wall component, this study has investigated the direct influence of MBL on* Candida in vitro. Candida (C.) albicans *and* C. glabrata *were* in vitro* exposed to different doses of recombinant human MBL for various time points to assess MBL influence on the production of hyphae and on the yeast forms. Moreover, the direct effect of MBL on the growth of* C. albicans *was measured by a cell proliferation assay. MBL induced agglutination of yeast forms as well as hyphal forms of* Candida *spp. and significantly reduced proliferation of* C. albicans in vitro*. MBL can be used as a potential antifungal candidate against* Candida* infection.

## 1. Introduction

Mannose-binding lectin (MBL), synthesized in the liver, is a member of a family of proteins called collectins, which is composed of collagenous domains linked to lectin domains. MBL is a large macromolecule that has a bouquet-like structure. The polypeptide chain of secreted MBL is 228 amino-acid long, not including the 20-residue signal peptide. The basic structural subunit of MBL is a homotrimer of MBL polypeptides, twisted in a triple helix. Each single polypeptide chain has four domains: (1) a 21-amino-acid N-terminal cysteine-rich (containing 3 cysteines) region involved in oligomerization by the formation of intra- and intersubunit disulphide bonds, (2) a 59-amino-acid collagen-like domain consisting of 20 tandem repeats of Glycine-Xaa-Yaa, where Xaa-Yaa indicate any amino acid (except repeat 8, which consists of only Glycine-Glutamine) that account for the long stalk of the molecule, (3) a 30-amino-acid *α*-helical, hydrophobic coiled-coil neck domain, which is crucial for initiating the oligomerization, and (4) a 188-amino-acid C-terminal carbohydrate recognition domain [1, 2]. Binding of MBL to pathogenic organisms leads to a change in the conformation of MBL multimer with subsequent activation of MBL-associated serine proteases (MASPs) and eventually initiation of complement lectin pathway [3].

Over the past four decades, many functions of MBL have been revealed. It is clear that MBL plays roles in complement activation, promotion of complement-independent opsonophagocytosis, modulation of inflammation, and recognition of altered self-structures and apoptotic cell clearance [4]. Low concentration of MBL is associated with increased susceptibility to infections [5].

The cell wall of* Candida* species (spp.) contains mannoproteins that display mannan in a variety of linkages. The mannan fraction of the cell wall is important for adhesion, cell wall integrity, and immune recognition and comprises up to 40% of the cell wall dry weight [6–8]. All of the major cell wall carbohydrate components of fungal walls serve as pathogen-associated molecular patterns, which are recognized by the innate immune system through pattern recognition receptors on the surface of immune effector cells [9]. Toll-like receptors and C-type lectin receptors, which are pattern recognition receptors, recognize molecular patterns on* Candida* cell wall, resulting in phagocytosis and killing of the invading fungus [10]. One of the C-type lectin receptors is the soluble, opsonic, multimeric MBL. MBL is able to trigger the complement cascade by recognizing and binding to carbohydrate moieties on the surface of microorganisms in general [11]. Besides, MBL binds with high-affinity to* Candida* spp. [12].

Different* Candida *spp.,* C. albicans *and* C. dubliniensis*, are associated with generation of hyphae. Hypha plays vital role in tissue invasion. Morphogenesis observed in* C. albicans* is induced by variations in temperature and pH and presence of serum [13]. Accordingly, most of pathogenicity of* C. albicans *owes to this transition [14]. On the contrary, under most conditions,* C. glabrata *exists only as yeast cells [15].

The aim of this study is to assess the* in vitro* capacity of MBL against yeast cells of* C. albicans *and* C. glabrata* as well as hyphal forms of* C. albicans*.

## 2. Materials and Methods

This study was conducted at Immunology Research Laboratory in Microbiology and Immunology Department, Faculty of Medicine, Zagazig University. This study was carried out in the period of July 2016 to August 2017.

### 2.1. Ethical Approval

This study was approved by the institutional review board, Faculty of Medicine, Zagazig University, Zagazig, Egypt.

*Candida Species. Candida *spp. were isolated from vaginal cultures of patients suffering from recurrent vulvovaginal candidiasis and presumptively identified by subculture on chromogenic agar medium* (CHROMagar™ Candida; Paris, France)*. Cultures were examined under light microscope to show the budding yeast cells with or without pseudohyphae, blastospores, and germ tubes [6, 16]. In addition, biochemical tests were studied using* Hi-Candida™ API* identification kit* (Biomereux, France)*. The isolated spp.,* Candida. (C.) albicans and C. glabrata*, were preserved on deep SDA and stored at 4°C for subsequent* in vitro* experiments.

*Candida *spp. maintained on deep SDA were allowed to grow overnight in brain-heart infusion broth* (Brain-Heart Infusion; Oxoid, UK)* at 37°C before use. These conditions allow* Candida *to grow as a >95% pure yeast phase population [17]. Immediately before each experiment,* Candida *spp., harvested by centrifugation, were washed 3 times with phosphate buffer saline (PBS)* (phosphate buffer saline 10x; Electron Microscopy Science, USA)* and resuspended to the appropriate concentration in PBS containing 1 mmol/L CaCl_2_ and 0.5 mmol/L MgCl_2_ (PBS^++^). Experiments were done in duplicate and repeated at least three times.

### 2.2. Influence of MBL on Hyphae Production and on Yeast Forms

*Candida *spp. (1 × 10^6^ cells/mL counted by hemocytometer) was incubated with MBL* (Recombinant Human MBL, R&D Systems, Minneapolis, USA)* in 100 *μ*L of PBS^++^, with and without 10% heat-inactivated fetal bovine serum (FBS)* (Fetal Bovine Serum; Sigma-Aldrich)* for* C. albicans* and with only 10% heat-inactivated FBS for* C. glabrata, *in 96-well microtiter plate at 37°C in 5% CO_2_ atmosphere* (Heraeus Hera cell)*.

Culturing at 37°C (5% CO_2_) in serum-rich medium stimulated* C. albicans *yeasts to germinate. The light microscopic analysis was used to assess MBL influence on yeast cells and on hyphae production. Human recombinant MBL was supplied lyophilized (50 *μ*g). It was reconstituted according to manufacturer's company protocol at a concentration of 100 *μ*g/mL with 0.5 mL sterile PBS. To determine the time-dependent factor for MBL influence,* Candida *yeast cells were incubated with MBL for various time points (30 min, 1 h, and 3 h). Besides, to determine the dose-dependent factor for MBL,* Candida *yeasts were incubated with various doses of MBL (0, 5, and 10 *μ*g/mL), since the average normal human MBL serum level is around 5 *μ*g/mL [17, 18].

### 2.3. Influence of MBL on the Growth of* Candida albicans*

Freshly grown* C. albicans* yeasts (2 × 10^7^ cells/mL) were incubated with MBL in a final volume of 100 *μ*L of PBS^++^ in Eppendorf tubes at 37°C in 5% CO_2_ atmosphere. To determine the time-dependent factor for MBL influence on* C. albicans *growth, cells were exposed to MBL for various time points (15 min and 30 min). Besides, to determine the dose-dependent factor for MBL,* C. albicans *yeasts were exposed to various doses of MBL (0, 5, and 10 *μ*g/mL) [17].

#### 2.3.1. Assessment of* Candida albicans* Growth

The yeast cells were washed with 1 mL of PBS followed by resuspension in RPMI (Roswell Park Memorial Institute) medium* (RPMI-1640; Sigma-Aldrich)* at 1 × 10^6^ cells/mL. A final volume of 100 *μ*L was transferred to a 96-well microtiter plate and incubated at 37°C in 5% CO_2_ atmosphere for 3 and 6 h. Wells containing RPMI medium alone and those containing 1 × 10^6^ cells/mL without previous exposure to MBL served as negative and positive control, respectively [17].

By using XTT-based cell proliferation assay* (XTT assay; Sigma-Aldrich)*, the growth of* C. albicans* was evaluated after 3 and 6 h of incubation. The XTT-based assay is a spectrophotometric method for estimating cell number based on the mitochondrial dehydrogenase activity in living cells. The key component is the sodium salt of XTT (2,3-bis[2-methoxy-4-nitro-5-sulfophenyl]-2H-tetrazolium-5-carboxyanilide inner salt). The mitochondrial dehydrogenases of viable cells reduce the tetrazolium ring of XTT, yielding an orange formazan derivative, which is water soluble. The absorbance of the resulting orange solution is measured spectrophotometrically [19]. XTT kit was supplied lyophilized (5 mg). It was reconstituted according to manufacturer's company protocol at a concentration of 1 mg/mL with 5 mL of sterile PBS. Warming the solution in a 56°C water bath helped to dissolve the dye. 20 *μ*L of the XTT solution was added to each well. The microtiter plates were then incubated in the dark at 37°C in 5% CO_2_ atmosphere for 2 h. A colorimetric change was then measured at a wavelength of 450 nm by using ELISA reader* (Stat Fax® 303 Plus)*.

### 2.4. Statistical Analysis

Quantitative data were represented as mean value ± 1 standard deviation (SD). *F* test was used for calculation of the mean difference between different groups. Least significance difference (LSD) test was used for multiple comparisons. Paired *t*-test was used for calculation of the mean difference within the same group at different time points. All analyses were 2-tailed. Results were considered statistically significant when* p* (probability) values were equal to or less than 0.05 at confidence interval (CI) 95%. All analyses were performed using Statistical Package for the Social Sciences software* (SPSS version 20, Inc., Chicago, IL, USA.)*.

## 3. Results

### 3.1. Influence of MBL on Hyphae Production and on Yeast Forms

**Figure 1:**
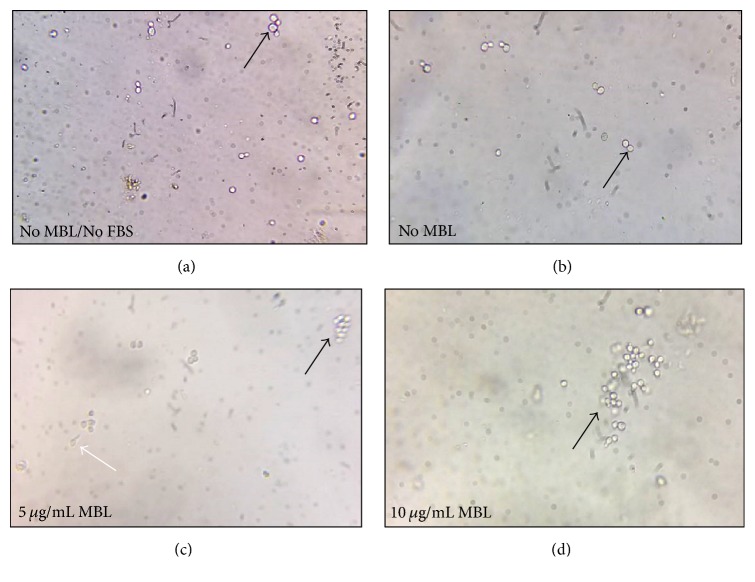
*MBL induced agglutination of Candida albicans after 30 min incubation*. (a) In absence of MBL and FBS, no agglutination is observed with yeast form of* C. albicans (Black arrow)*. (b) In absence of MBL and presence of 10% FBS, no agglutination is observed with yeast phase of* C. albicans (Black arrow)*. (c) and (d) In presence of 5 and 10 *μ*g/mL MBL with 10% FBS, agglutination of yeast phase of* C. albicans* is started to be observed* (Black arrow)*. Germination of hyphae starts to occur* (White arrow)*.

**Figure 2:**
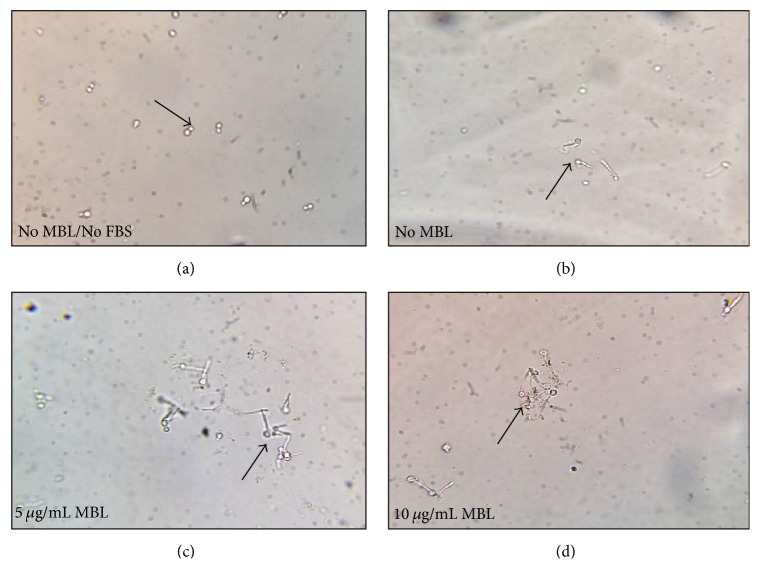
*MBL induced agglutination of Candida albicans after 1 h incubation*. (a) In absence of MBL and FBS, no agglutination is observed with yeast form of* C. albicans (Black arrow)*. (b) In absence of MBL and presence of 10% FBS, no agglutination only germination of hyphae is observed* (Black arrow)*. (c) and (d) In presence of 5 and 10 *μ*g/mL MBL with 10% FBS, agglutination of germinated hyphae of* C. albicans* is started to be observed* (Black arrow)*.

**Figure 3:**
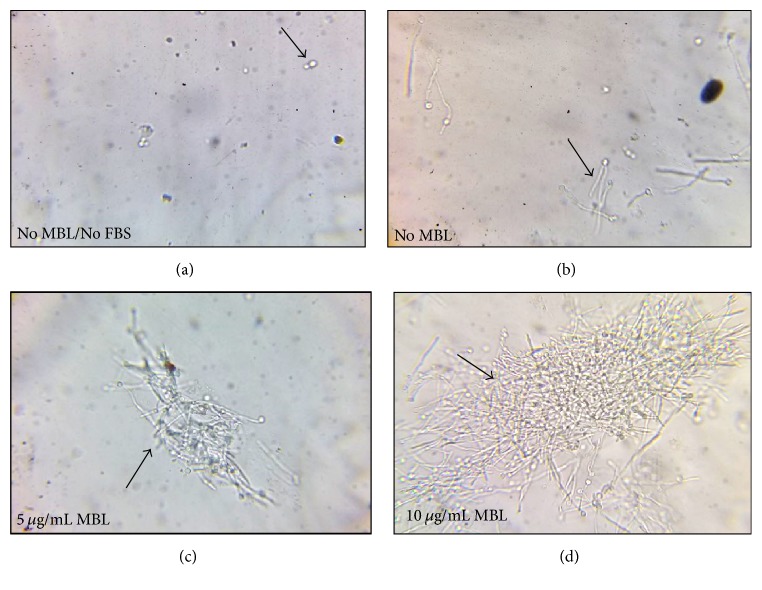
*MBL induced agglutination of Candida albicans after 3 h incubation*. (a) In absence of MBL and FBS, no agglutination is observed with yeast form of* C. albicans (Black arrow)*. (b) In absence of MBL and presence of 10% FBS, no agglutination only elongation of hyphae is observed* (Black arrow)*. (c) and (d) In presence of 5 and 10 *μ*g/mL MBL with 10% FBS, increased agglutination and elongation of hyphae of* C. albicans* are observed* (Black arrow)* The effect is more potentiated with 10 *μ*g/mL MBL when compared to 5 *μ*g/mL.

**Figure 4:**
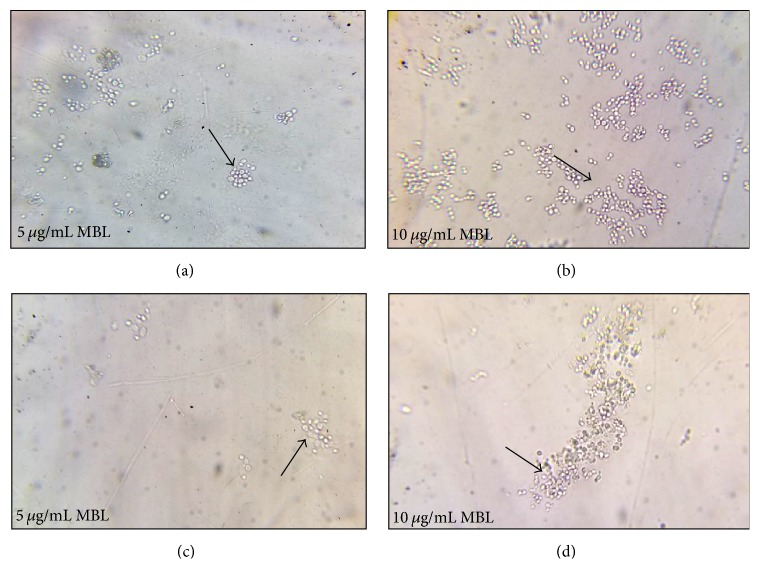
*MBL induced agglutination of yeast cells of Candida species after 3 h incubation.* (a) and (b) In presence of 5 and 10 *μ*g/mL MBL without FBS, agglutination of* C. albicans* is observed. (c) and (d) The same effect of 5 and 10 *μ*g/mL MBL is also observed with* C. glabrata*. The effect is more potentiated with 10 *μ*g/mL MBL* (Black arrows)* when compared to 5 *μ*g/mL MBL* (Black arrows)*.

Light microscopic analysis revealed that MBL had no influence on the germination of* C. albicans *yeasts.* C. albicans* yeasts in the presence or absence of MBL germinated and formed hyphae within 3 h incubation. However, in the presence of MBL, agglutination of hyphae was observed. Moreover, in the presence of MBL, agglutination was observed when (1) yeast phase of* C. albicans *was incubated with 10% FBS, (2) yeast forms of* C. albicans* were incubated without FBS, and (3) yeast forms* C. glabrata *were incubated with 10% FBS. When the dose-dependent factor was evaluated, agglutination of both hyphae and yeast cells was markedly observed with 10 *μ*g/mL MBL compared to 5 *μ*g/mL MBL. When the time-dependent factor was evaluated, agglutination of hyphae markedly increased as more hyphae were induced and elongated over different time points: 30 min, 1 h, and 3 h incubation. Besides, agglutination of yeast cells was markedly increased over different time points, 30 min, 1 h, and 3 h incubation, as shown in Figures 1–4.

### 3.2. Influence of MBL on the Growth of* Candida albicans* after 3 h Incubation

**Figure 5:**
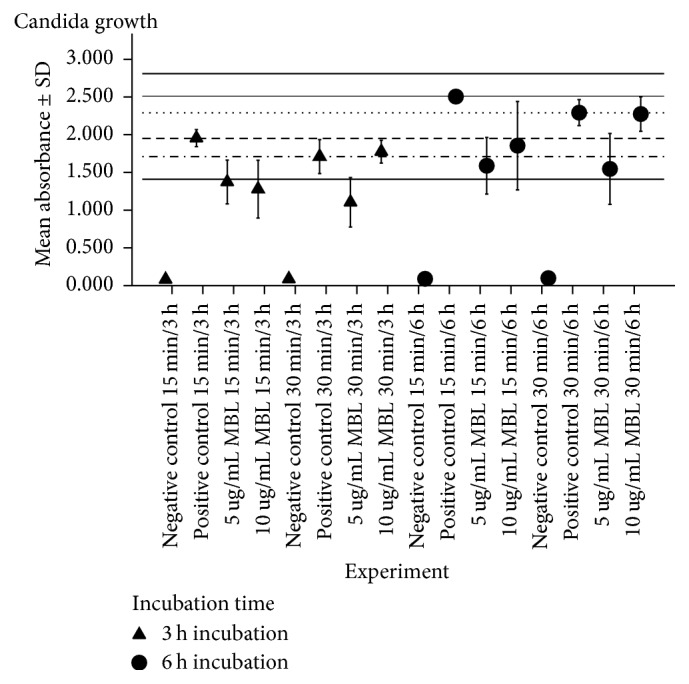
*Summary of direct influence of MBL on Candida albicans growth in vitro*. Dashed line (- - - - - - -) represents mean absorbance of positive control (1.95 ± 0.11) after 15 min exposure to 0 *μ*g/mL MBL and 3 h incubation period. Dot-dash line (· –·–·–·– · –) represents mean absorbance of positive control (1.71 ± 0.23) after 30 min exposure to 0 *μ*g/mL MBL and 3 h incubation period. Continuous line (—) represents mean absorbance of positive control (2.51 ± 0.04) after 15 min exposure to 0 *μ*g/mL MBL and 6 h incubation period. Dotted line (………) represents mean absorbance of positive control (2.29 ± 0.17) after 30 min exposure to 0 *μ*g/mL MBL and 6 h incubation period. Data are plotted as mean absorbance ± SD of 3 separate experiments. Absorbance is at 450 nm.

When the direct influence of MBL on the growth of* C. albicans *by XTT assay was estimated after 3 h incubation period, there was statistically significant reduction in growth when* C. albicans* was exposed to 5 and 10 *μ*g/mL MBL for 15 min and to 5 *μ*g/mL MBL for 30 min compared to positive control (MBL = 0 *μ*g/mL) (*p* = 0.013, *p* = 0.005, and *p* = 0.004, resp.). However, there was no reduction in growth after exposure to 10 *μ*g/mL MBL for 30 min compared to positive control (*p* = 0.700). When the dose-dependent factor was evaluated, there was no statistically significant difference in growth after exposure to 5 and 10 *μ*g/mL MBL for 15 min (*p* = 0.631). However, after 30 min exposure, there was statistically significant reduction in growth with 5 *μ*g/mL MBL compared to 10 *μ*g/mL MBL (*p* = 0.002). When the time-dependent factor was evaluated, there was no statistically significant difference in growth between 15 and 30 min exposure to both 5 and 10 *μ*g/mL MBL (*p* = 0.264 and *p* = 0.123, resp.) as demonstrated by Table 1 and Figure 5.

### 3.3. Influence of MBL on the Growth of* Candida albicans* after 6 h Incubation

When the direct influence of MBL on the growth of* C. albicans *by XTT assay was estimated after 6 h incubation period, there was statistically significant reduction in growth when* C. albicans *was exposed to 5 and 10 *μ*g/mL MBL for 15 min and to 5 *μ*g/mL MBL for 30 min compared to positive control (MBL = 0 *μ*g/mL) (*p* = 0.007, *p* = 0.036 and *p* = 0.006, resp.). However, the reduction of growth was not statistically significant when* C. albicans *was exposed to 10 *μ*g/mL MBL for 30 min compared to positive control (*p* = 0.934). When the dose-dependent factor was evaluated, there was statistically significant reduction in growth after 30 min exposure to 5 *μ*g/mL MBL compared to 10 *μ*g/mL MBL; however, such reduction was not statistically significant after 15 min exposure, (*p* = 0.007, *p* = 0.346, resp.). When the time-dependent factor was evaluated, there was no statistically significant difference in growth between 15 and 30 min exposure to both 5 and 10 *μ*g/mL MBL (*p* = 0.922 and *p* = 0.198, resp.) as demonstrated by Table 2 and Figure 5.

## 4. Discussion

For more than a decade, the potential of MBL as a therapeutic agent has been proposed [20]. MBL replacement therapy was previously investigated in patients with recurrent erythema multiform and severe cystic fibrosis via infusion of fresh frozen plasma containing MBL, resulting in clinical improvement of the patients [21, 22]. In this context, the present study has investigated the direct influence of MBL on* C. albicans *and* C. glabrata in vitro*.

This study demonstrates that MBL recognized* Candida* spp. and induced their agglutination in yeast form as well as upon induction of hyphae. Nevertheless, MBL was unable to inhibit the transition of* C. albicans* from the yeast phase to hyphal phase, since* C. glabrata* grows strictly in pure yeast form under most conditions [15]. Besides, MBL induced agglutination was both time- and dose-dependent. A possible explanation is that long incubations were associated with more hyphal outgrowth with a subsequent increase in MBL-ligand expression on the growing hyphae. Similarly, budding of the yeast cells could enhance the chance of MBL-ligand expression.

These findings agree with the study of Lillegard and his colleagues who found that MBL binds extensively to both hyphae and budding yeast cells [8]. Moreover, the previous report of Ip and Lau suggested that steric hindrance by MBL can prevent the spread of the virulent forms of* Candida* by blocking the receptors. However, Ip and Lau found that MBL induced agglutination only with hyphal forms of* C. albicans *but not with yeast forms [17].

The present study has reported significant suppression in the growth of* C. albicans* during an incubation period for up to 6 hours. This significant suppression was observed with exposure to 5 *μ*g/mL MBL for 15 and 30 min, while 10 *μ*g/mL MBL induced significant suppression with exposure for 15 min but not 30 min. MBL induced growth suppression was not time-dependent; however, it was dose-dependent only with 30 min exposure. The 5 *μ*g/mL MBL is quite close to the average normal human MBL serum level [18]. Therefore, it seems that 5 *μ*g/mL MBL was optimum and more effective than 10 *μ*g/mL MBL. Moreover, MBL-ligand binding, like any macromolecules, is governed by the dissociation constant, and the affinity of binding is much influenced by hydrogen bonding, electrostatic interaction, and hydrophobicity of the cell surface clustering of the ligands. In addition, multivalent binding results in clustering of the receptors [23].

This work agrees with the study of Ip and Lau. They observed that MBL induced a significant reduction in the growth of* C. albicans *when added to heat-inactivated MBL-deficient serum, a situation in which complement activation would not occur. This suggested that MBL possesses an intrinsic mechanism through which it inhibits the fungal growth. However, the maximum inhibitory effect was observed with unheated MBL-deficient serum demonstrating the importance of lectin pathway in inhibiting the growth of* C. albicans *[17]. When mice, treated with intravenous MBL, were challenged with* C. albicans*, prolonged survival of mice was significantly observed [8]. It is also possible that MBL induced agglutination of yeast cells could limit the availability of the nutrients to them resulting in growth inhibition. Consistently, intravaginal administration of recombinant human MBL coupled to itraconazole in* MBL* gene knockout mice with* C. albicans *vaginitis resulted in 3-fold clearance of yeast compared to itraconazole alone [24]. Although its clinical efficacy has not been clearly evidenced, no side effects of exogenous MBL administration were identified, and some clinical benefits were apparent [25]. Therefore, the present study strongly recommends the potential usefulness of this approach and its extension to large-scale randomized clinical trials; hence, this would provide a solid evidence concerning the physiological significance of MBL against resistant or recurrent* Candida *infections, particularly in MBL-deficient subjects.

## 5. Conclusion

Recombinant human MBL can induce agglutination of* C. albicans *and* C. glabrata *yeast cells and hyphal forms of* C. albicans. *Moreover, it can significantly reduce the growth of* C. albicans in vitro. *These effects could nominate MBL as a potential therapeutic agent against* Candida* infection.

## Figures and Tables

**Table 1:** Direct influence of MBL on the growth of *Candida albicans* measured by XTT assay after 3 h incubation period.

MBL concentration	0 *µ*g/mL	5 *µ*g/mL	10 *µ*g/mL	Test of significance	*p* value	LSD *p* value
15 min exposure to MBL						
Absorbance Mean ± SD	1.95 ± 0.11	1.37 ± 0.29	1.28 ± 0.38	*F test* **21.398**	**<0.00**1^*∗*^	0.013^*∗∗*1^ 0.005^*∗∗*2^ 0.631^*∗∗*3^
30 min exposure to MBL						
Absorbance Mean ± SD	1.71 ± 0.23	1.10 ± 0.33	1.78 ± 0.15	*F test* **28.583**	**<0.00**1^*∗*^	0.004^*∗∗*1^ 0.700^*∗∗*2^ 0.002^*∗∗*3^
Between different MBL exposure time points (15 and 30 min)						
Test of significance		Paired *t*-test				
		**t** = 1.370	**t** = −2.128			
*p* value		0.264^*∗∗*4^	0.123^*∗∗*5 ^			

^*∗*^Significant difference; ^*∗∗*1^*p* value between 0 and 5 *µ*g/mL MBL; ^*∗∗*2^*p* value between 0 and 10 *µ*g/mL MBL; ^*∗∗*3^*p* value between 5 and 10 *µ*g/mL MBL; ^*∗∗*4^*p* value between 15 and 30 min exposures to 5 *µ*g/mL MBL; ^*∗∗*5^*p* value between 15 and 30 min exposures to 10 *µ*g/mL MBL.

**Table 2:** Direct influence of MBL on the growth of *Candida albicans* measured by XTT assay after 6 h incubation period.

MBL concentration	0 *µ*g/mL	5 *µ*g/mL	10 *µ*g/mL	Test of significance	*p* value	LSD *p* value
15 min exposure to MBL						
Absorbance Mean ± SD	2.51 ± 0.04	1.59 ± 0.37	1.86 ± 0.59	*F test* **18.197**	**<0.00**1^*∗*^	0.007^*∗∗*1^ 0.036^*∗∗*2^ 0.346^*∗∗*3^
30 min exposure to MBL						
Absorbance Mean ± SD	2.29 ± 0.17	1.55 ± 0.47	2.27 ± 0.23	*F test* **29.076**	**<0.00**1^*∗*^	0.006^*∗∗*1^ 0.934^*∗∗*2^ 0.007^*∗∗*3^
Between different MBL exposure time points (15 and 30 min)						
Test of significance		Paired *t*-test				
		**t** = 0.106	*t* = −1.647			
*p* value		0.922^*∗∗*4^	0.198^*∗∗*5^			

^*∗*^Significant difference; ^*∗∗*1^*p* value between 0 and 5 *µ*g/mL MBL; ^*∗∗*2^*p* value between 0 and 10 *µ*g/mL MBL; ^*∗∗*3^*p* value between 5 and 10 *µ*g/mL MBL; ^*∗∗*4^*p* value between 15 and 30 min exposures to 5 *µ*g/mL MBL; ^*∗∗*5^*p* value between 15 and 30 min exposures to 10 *µ*g/mL MBL.
